# Health-Risk Behaviours and Injuries among Youth and Young Adults in Chiang Mai, Thailand: A Population-Based Survey

**DOI:** 10.3390/ijerph17103696

**Published:** 2020-05-24

**Authors:** Apichai Wattanapisit, Wichuda Jiraporncharoen, Kanokporn Pinyopornpanish, Surin Jiraniramai, Kanittha Thaikla, Chaisiri Angkurawaranon

**Affiliations:** 1 School of Medicine, Walailak University, Nakhon Si Thammarat 80161, Thailand; apichai.wa@gmail.com; 2 Department of Family Medicine, Faculty of Medicine, Chiang Mai University, Chiang Mai 50200, Thailand; wichudaj131@gmail.com (W.J.); kpinyopo@gmail.com (K.P.); sjiranir@gmail.com (S.J.); 3 Research Institute for Health Sciences, Chiang Mai University, Chiang Mai 50200, Thailand; kthaikla@hotmail.com

**Keywords:** alcohol, gambling, health-risk behaviours, injuries, smoking, young adults, youth

## Abstract

This study aimed to identify the prevalence of health-risk behaviours (alcohol use, tobacco smoking and gambling) and the associations between health-risk behaviours and injuries among youth (15–24 years) and young adults (25–39 years). A multi-stage cluster sampling survey was conducted in Chiang Mai, Thailand. The associations between health-risk behaviours and injuries were analysed using logistic regression and adjusted for potential confounders. Sample weights were applied in all analyses. Six-hundred-and-thirty participants were included. Fifty-three percent of males and 12.3% of females drank in the past three months. Smoking in the past three months was higher among males (38.5%) than females (0.7%). About a quarter of men and a fifth of the women had gambled in the past year. A total of 6.4% of males and 4.8% of females sought medical attention in the past year due to injuries. Compared to those without any of the three health-risk behaviours, the odds ratio for injuries requiring medical attention was 3.81 (95% CI: 1.33 to 10.90, *p* = 0.013) for those with two health-risk behaviours and 13.8 (95% CI: 4.24 to 45.10, *p* < 0.001) for those with all three health-risk behaviours. Injury prevention policies may need to incorporate interventions designed to assess multiple health-risk behaviours.

## 1. Introduction

In 2017, injuries and violence caused more than 4.4 million deaths globally, an increase of 2.3% from 2007 [1]. According to the Global Burden of Disease Study 2017, unintentional injuries accounted for approximately 1.8 million deaths, followed by self-harm and interpersonal violence (1.3 million deaths) and transport injuries (1.3 million deaths) [1]. Additionally, injuries contributed to 57.7 million years lived with disability [2]. In Thailand, a study published in 2012 estimated that one-fifth of Thai adults had at least one injury in the past 12 months [3]. Road traffic injuries alone contributed to 673,000 years lived with disability and more than ten thousand deaths each year in Thailand [4,5]. Moreover, 60% of injury mortality was attributed to non-transport injuries [6]. 

Injuries are a major public health concern among youth and young adults as this age group is likely to have several behavioural risk factors, which may be associated with a higher risk of injuries [7,8]. Major health-risk behaviours among youth and young adults that can contribute to injuries include the use of tobacco, alcohol and other substances, gambling and unhealthy lifestyles [9]. For example, dos Santos Silva et al. reported that alcohol consumption and cigarette smoking can increase the risk of violence among young people by 3.4 times and 3.8 times, respectively [7]. Other studies also confirmed that alcohol use [10,11] and tobacco smoking [12] are behavioural risk factors associated with increased injuries. A systematic review and meta-analysis presented a dose–response relationship of alcohol consumption and both motor vehicle (increased 1.2 times for every 10-g increase in alcohol consumption) and non-motor vehicle injuries (increased 1.3 times for every 10-g increase in alcohol consumption) [11]. Alcohol affects psychomotor abilities and relates to aggression and inhibitory control [13,14,15]. While the association between smoking and injuries could be due to its correlation with alcohol use, there is evidence that smoking could also be an independent risk factor for motor-vehicle-related injuries [16]. While the potential mechanism remains unclear, it has been hypothesised that smoking could increase distractions to drivers or that smoking may cause physiological and/or neurological responses that may interfere with driving [17]. Besides distractibility and intoxication, the associations between smoking with injuries could be due to smoking-associated medical conditions and personality or behavioural characteristics [18]. A less commonly examined behavioural factor associated with injuries is gambling. There are many potential mechanisms linking gambling with injuries [19]. For example, it could be associated through its correlation with substance use since problematic gamblers tend to have high rates of tobacco and alcohol use [20]. Injuries from interpersonal harm and violence are also associated with gambling [21]. For example, problematic gambling may cause financial problems that may lead to relationship problems, illegal problems or criminal problems, which increase the risk of physical injuries [19]. Several studies found that gambling is also associated with aggressive behaviours, such as physical fighting, weapon carrying and violence [22,23,24]. The psychosocial effects of problematic gambling may also lead to depression, mental health problems and subsequently put gamblers at a higher risk of physical injuries from personal and interpersonal harm, such as domestic violence, abuse and accidents [19]. The Thai National Mental Health survey 2013 estimated that almost 10% of the population between the ages of 18 to 34 have gambled at least once per week for 6 months or more consecutively [25]. The same study also identified links between gambling disorders and mental health problems, such as depression and drug dependence.

The study of injuries and violence is an important research area of adolescent health in low- and middle-income countries (LMICs), which is a leading cause of global mortality [26,27]. The estimates of alcohol and tobacco use, gambling and injuries among youth and young adults in Thailand have been reported [25,28,29,30]. However, like other LMIC settings, the relationship between multiple health-risk behaviours and injuries has not been commonly explored. The present study aimed to (i) estimate the prevalence of three health-risk behaviours (alcohol use, tobacco smoking and gambling) and injuries among youth and young adults from Thailand and (ii) explore the associations between health-risk behaviours and both intentional and unintentional injuries. We hypothesised that a combination of multiple health-risk behaviours could potentially lead to a higher risk of injuries among young people [8]. 

## 2. Material and Methods

### 2.1. The Dataset

This study utilised a subset of a dataset from a population-based behavioural health survey conducted in Chiang Mai, a province of Northern Thailand. The survey was designed to be representative of the population (ages 15 to 64) living in Chiang Mai, estimated at 1.6 million people. The details of the methods used have been published [31]. In short, a two-stage stratified cluster-sampling survey using probability sampling proportional to size, such that the ratio of urban and rural units was 2:1, was conducted in 2014. The first primary unit for sampling was the enumeration areas as defined by the National Statistical Office of Thailand. In the second stage, 20 households from each enumeration area were systematically sampled. Trained field staff conducted face-to-face structured interviews assessing multiple behavioural health issues, including substance use, sexual health and physical activity [32,33]. The total sample size was 1744 and the sample represented the source population well in terms of key demographics (age and gender distributions) [33]. The target population in this study were the youth and young adults aged 15–39 years, which reduced the sample size for analysis to 630.

### 2.2. Variables and Measurements 

The sociodemographic variables included (i) age group: youth (15–24 years) and young adults (25–39 years); (ii) gender: female and male; (iii) marital status: single, married/partnered and divorced/separated/widowed; (iv) highest education: primary, early secondary, late secondary and bachelor’s and higher; and (v) living location: rural and urban. 

Health-risk behaviours explored in this study were alcohol use, tobacco smoking and gambling. Alcohol use and tobacco smoking were collected based on the Alcohol, Smoking and Substance Involvement Screening Test (ASSIST), which was developed by the World Health Organization (WHO) [34], and items used in the fifth Thailand National Health Examination Survey [35]. Participants were asked whether they had used alcohol in their lifetime, whether they had used alcohol in the past three months and whether they had heavy alcohol use in the past 30 days. Heavy alcohol use was defined as having at least six standard drinks per sitting, where one standard drink was defined as 10 grams of pure alcohol [36]. Alcohol use was then classified into four categories: (i) had never used alcohol in their lifetime, (ii) had used alcohol but not in the past three months, (iii) had used alcohol in the past three months but no heavy alcohol use in the past 30 days and (iv) heavy alcohol use in the past 30 days. Tobacco smoking was divided into three categories: (i) had never smoked in their lifetime, (ii) past smoker (had smoked in their lifetime but not in the past three months), and (iii) smoked in the past three months. The survey on gambling was collected by using a questionnaire adapted from a survey on gambling among Thai college students [37]. Both legal and illegal gambling was assessed. In Thailand, the main form of legal gambling was the lottery, which is popular among both men and women [25]. Common illegal gambling among both men and women in Thailand includes the underground lottery and playing games of cards or dice for money. Additionally, online gambling and sports betting are also common among men in Thailand [25]. We categorised gamblers into four groups: (i) had never gambled, (ii) had legally gambled in the past 12 months, (iii) had illegally gambled in the past 12 months and (iv) had legally gambled and illegally gambled in the past 12 months.

For injuries, both intentional and unintentional injuries in the past 12 months were considered. In the questionnaire, participants were asked about five main types of injuries: (i) road-traffic-related injuries; (ii) physical injuries caused by gunshots or firearms; (iii) physical injuries from other types of objects/weapons; (iv) non-firearm/non-weapon-related physical injuries, such as from fights/altercations, falls or accidents; and (v) self-inflicted injuries. The variables of interest regarding injuries were: (i) any injuries requiring medical attention, (ii) any injuries requiring hospital admission, and (iii) injuries from a road traffic accident requiring medical attention.

### 2.3. Statistical Analysis 

The sociodemographic data were described using frequencies and percentages. The prevalence and 95% confidence interval (95% CI) of alcohol use, tobacco smoking, gambling and injuries by age group and gender were calculated. Logistic regression was used to compare the prevalence of each health-risk behaviour between genders. The associations between health-risk behaviours and injuries were also identified by using logistic regression. In the first regression model (model 1), each health-risk behaviour was adjusted for key sociodemographic factors previously identified in the literature as potential confounders in the association between health-risk behaviours and injuries. These key sociodemographic factors consisted of age, gender, living location and education level [3,25]. The second model (model 2) further included all three health-risk behaviours as covariates in the model. Adjusted Wald tests were used to test for associations between each health-risk behaviour and injury. To account for the multi-testing of three outcome measures of injury for each health-risk behaviour, Bonferroni corrections were used [38]. To keep the overall alpha level at 5%, the corrected alpha level for each test was set at 1.7% (0.05/3). Interactions between gender and health-risk behaviors were also explored. 

To explore whether an increasing number of health-risk behaviours would be associated with an increased risk of injuries, categories within each health-risk behaviour were collapsed into a binary variable based on the association presented in model 1. Within each variable, categories that demonstrated a similar risk of injuries to the reference category would be coded as “0”, while categories demonstrating an increased risk of injuries compared to the reference category would be coded as “1”. Logistic regression was then used to analyse the association between the number of health-risk behaviours: 0 (no risk factors), 1 (one risk factor), 2 (two risk factors) and 3 (three risk factors), and each of the three injury outcomes of interest. An adjusted Wald test was used to correct for pair-wise testing. Using Bonferroni correction for the three outcome measures of injury tested [38], the corrected alpha level was set at 1.7%. Sample weights were applied in all analyses and operated using the “*svy*” command in STATA version 13 (StataCorp, College Station, TX, USA) to account for the complex cluster design.

### 2.4. Ethics Approval

The study protocol was approved by the Research Ethics Committee, Faculty of Medicine, Chiang Mai University (No. 62/2014). Participation in the study was entirely voluntary. Written informed consent was taken from participants or their legal guardians (for participants aged under 18 years) before the interviews.

## 3. Results

### 3.1. Study Participants

Of the 630 participants, 35.6% were less than 25 years of age, 45.7% were single, 40.6% completed late secondary education, and 65.9% lived in rural areas (Table 1).

### 3.2. Prevalence of Health-Risk Behaviours and Injuries

Overall, the prevalence of health-risk behaviours, including alcohol use, tobacco smoking and gambling, was higher among young adults (compared to youth) and males (compared to females). A total of 72.8% of males had used alcohol in their lifetime. Fifty-three percent of males and 12.3% of females drank in the past three months. Males were more likely to be heavy alcohol drinkers than females (20.3% vs. 2.1%). Forty-seven percent of males and 2.8% of females had smoked in their lifetime. Smoking in the past three months was significantly higher among males (38.5%) than females (0.7%). About one-third of males and about one-quarter of females had gambled in their lifetime, while a quarter of men and a fifth of women had gambled within the past 12 months. Overall, legal gambling was more favoured compared to illegal gambling in both males and females (Table 2).

**Figure 1:**
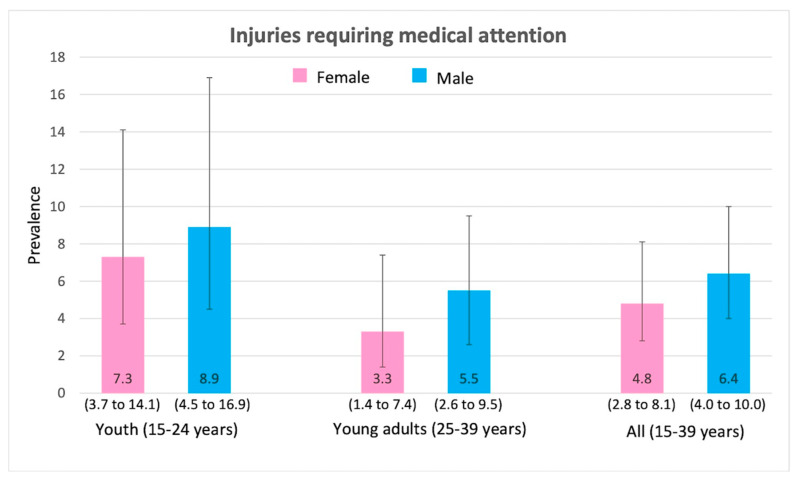
Prevalence (and 95% CI intervals) of injuries requiring medical attention by age group and gender.

**Figure 2:**
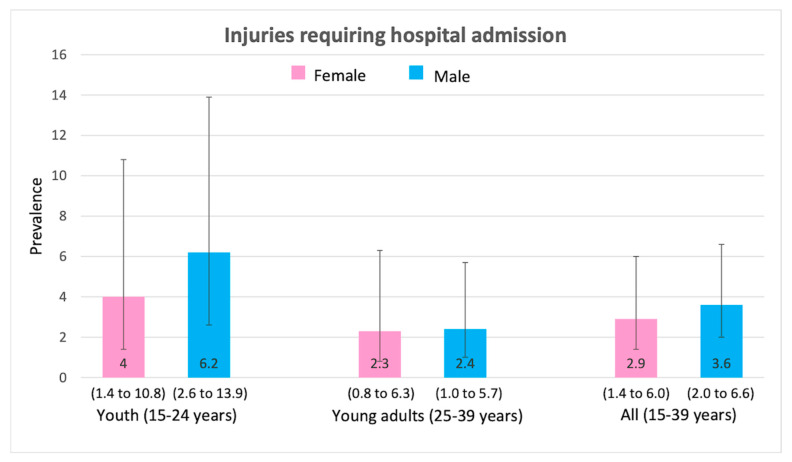
Prevalence (and 95% CI intervals) of injuries requiring hospital admission by age group and gender.

**Figure 3:**
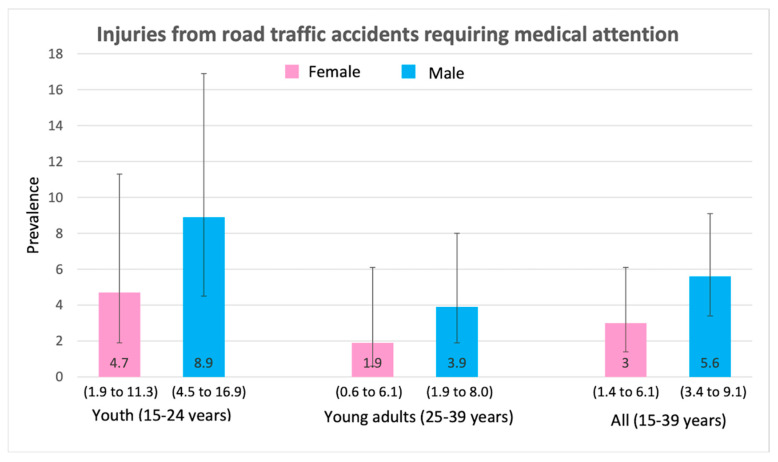
Prevalence (and 95% CI intervals) of injuries from road traffic accidents requiring medical attention by age group and gender.

Injuries were slightly higher in males (compared to females). However, injuries were lower in young adults (ages 25–39) for both males and females when compared to youth (ages 15–24). Figure 1 shows that 6.4% of males and 4.8% of females sought medical attention in the past 12 months due to injuries. As a result, 3.6% of males and 2.9% of females were admitted to hospitals in the past 12 months (Figure 2). A total of 5.6% of males and 3.0% of females needed medical attention due to road traffic injuries (Figure 3).

### 3.3. Health-Risk Behaviours and Injuries

There was no significant evidence for interactions between gender and health-risk behaviours as the association between health-risk behaviours and injuries did not seem to differ by gender. Adjusting for potential confounders, there was evidence to suggest that smoking (*p* = 0.011) was associated with injuries requiring medical attention. Alcohol use (*p* = 0.078) and gambling (*p* = 0.116) also had a positive association with injuries requiring medical attention but did not achieve statistical significance. There were consistently positive associations between each health-risk behaviour and other types of injuries, but they also did not achieve statistical significance (Table 3). 

However, the study demonstrated that those with a higher number of health-risk behaviours also had higher odds of receiving injuries (Table 4). Adjusting for age, gender, education and living location, the odds ratio for injuries requiring medical attention was 3.81 (95% CI: 1.33 to 10.90, *p* = 0.013) for those with two health-risk behaviours (compared to those without any of the three risk behaviours). For those with all three health-risk behaviours, the odds ratio (compared to those without any of the three health-risk behaviours) was 13.8 (95% CI: 4.24 to 45.10, *p* < 0.001). The same pattern emerged for injuries requiring hospital admission and injuries from road traffic injuries requiring medical attention. The higher the number of health-risk behaviours, the higher the odds of receiving injuries when compared to those without any of the three health-risk behaviours (Table 4).

## 4. Discussion

The study investigated three common health-risk behaviours (alcohol use, smoking, and gambling) and injuries among youth and young adults in Chiang Mai, Thailand. Compared to youth (15–24 years), young adults (25–39 years) had a higher prevalence of alcohol use, smoking, and gambling. On the other hand, the youth had a higher prevalence of injuries. Regarding health-risk behaviours, alcohol use and tobacco smoking were common health-risk behaviours among males. Males also had a higher rate of injuries than females. However, the differences in the prevalence of injuries between genders were small. Compared to displaying no health-risk behaviour, the results supported the fact that having a combination of health-risk behaviours was associated with higher odds of receiving injuries. 

The interpretation of the study needs to take into account its strengths and limitations. The strengths of the study were that it was a representative survey of a population in Chiang Mai province, Thailand, and the analysis accounted for the clustering of data. However, there were some limitations. First, the findings may not be generalisable to other populations. Second, the cross-sectional design could not determine the temporality of health-risk behaviours and injuries. The survey relied on self-reported behaviours and injuries, which may lead to under-reporting of these health-risk behaviours and outcomes. These imprecisions in measurements are likely to dilute any potential associations. Not all risk factors potentially associated with health-risk behaviours and injuries, such as psychiatric morbidities and school/social exclusion [39], were assessed; thus, there was potential for other unmeasured and residual confounding. The analyses might be unable to detect some outcomes of interest due to the low prevalence of injuries. Lastly, we tested several associations that could lead to alpha-error inflation. However, we have tried to correct for multiple comparisons in our analyses. 

Our findings are supported by the literature. Evidence supports that males are more likely than females to consume alcohol [40,41,42], smoke cigarettes [43,44,45] and gamble [46,47]. Moreover, our findings showed males were more likely to be injured and required more healthcare services. A higher rate of injuries requiring medical attention and hospital admission among males might reflect more severe injury outcomes. The WHO also showed that males were more at risk of serious outcomes from injuries and violence [48]. This reflects the importance of focusing on men’s health since men may have different health issues than women [49]. However, the differences between the prevalence of injuries between males and females were much smaller compared to the differences in health-risk behaviours. The fact that females may have a similar risk of injuries despite a lower prevalence of health-risk behaviours suggests that females could be more vulnerable to injuries. For example, evidence has suggested that females are more likely to be victims of physical violence [50]. In the United States, fatal road traffic injuries also seem to be increasing among females, despite similar risk behaviours and driving patterns [51]. This suggests that injury prevention programmes may require different focuses between genders. Males’ interventions need to focus on a reduction of health-risk behaviours, while females should be empowered to reduce vulnerability to injuries. 

We found that young adults (ages 25–39) were more likely to drink alcohol, smoke cigarettes and gamble than youths (ages 15–24). Other literature has also shown that during the transition from adolescence to young adulthood, there were significant increases in health-risk behaviours [52]. Our study suggests that youth were more at risk of injuries requiring medical attention or hospital admission than young adults. This is also in line with the WHO’s report, which stated that three out of the top four leading causes of death among people aged 15–29 years were road traffic injuries and intentional injuries (suicide and homicide) [48]. 

Previous literature suggested that alcohol use, smoking and gambling were associated with injuries [10,16,21,53]. While many external socioenvironmental factors can influence a person’s behaviour [54], at an individual level, gambling and substance use can be conceptualised as expressions of impaired behavioural impulse control [22] associated with increased aggressiveness, risk-taking, impulsivity and potential mental health problems [55,56,57], which subsequently may lead to intentional and unintentional injuries [19]. The evidence shows that young people in the UK tend to have more than one health-risk behaviour [58]. Moreover, similar to the results found in our study, a study investigating health-risk behaviours and injuries among youth in 12 countries found that the odds of injuries increased with an increased number of health-risk behaviours [8].

## 5. Conclusions

Despite its limitations, this study offers insights into three health-risk behaviours and injuries among youth and young adults from Thailand. Most injury prevention programmes focus on traffic-related injuries and alcohol use [59]. However, as these health-risk behaviours may also cluster and lead to increasing the risk of injuries, interventions may need to be designed to assess multiple health-risk behaviours and provide tailored feedback and interventions [60,61,62].

## Figures and Tables

**Table 1:** Sociodemographic characteristics (aged 15–39 years).

Characteristics	Number in Sample
	*n* (%)
**Total participants**	630
**Age group**	
Youth (15–24 years)	224 (35.6)
Young adults (25–39 years)	406 (64.4)
**Gender**	
Female	329 (52.2)
Male	301 (47.8)
**Marital status**	
Single	288 (45.7)
Married/partnered	306 (48.6)
Divorced/separated/widowed	36 (5.7)
**Highest education**	
None or primary	76 (12.1)
Early secondary	113 (17.9)
Late secondary	256 (40.6)
Bachelor’s or higher	185 (29.4)
**Living location**	
Urban location	215 (34.1)
Rural location	415 (65.9)

**Table 2:** Prevalence and 95% confidence interval of health-risk behaviours by age group and gender in Chiang Mai.

Health-Risk Behaviours By Age Group and Gender	Youth		Young Adults		All	
	(15–24 Years)		(25–39 Years)		(15–39 Years)	
	Female	Male	Female	Male	Female	Male
Number in sample	127	97	202	204	329	301
**Alcohol Use (prevalence and 95% confidence interval)**						
Had used alcohol in their lifetime	18.2	58.0 *	29	80.2 *	24.9	72.8 *
	(12.3 to 26.1)	(47.3 to 48.0)	(22.8 to 36.2)	(73.9 to 85.2)	(20.2 to 30.1)	(67.2 to 77.7)
Alcohol use in the past 3 months	8.1	39.4 *	14.7	60.0 *	12.3	53.0 *
	(4.4 to 14.4)	(29.7 to 50.0)	(10.2 to 20.6)	(52.7 to 66.7)	(8.9 to16.3)	(47.1 to 58.9)
Heavy alcohol use in the past 30 days	2.4	12.6 *	1.9	24.2*	2.1	20.3 *
	(0.7 to 7.5)	(7.9 to 22.0)	(0.7 to 5.1)	(18.5 to 31.0)	(1.0 to 4.4)	(15.9 to 25.6)
**Tobacco Smoking (prevalence and 95% confidence interval)**						
Had smoked in their lifetime	0.8	27.2 *	4.2	56.9 *	2.8	47.0 *
	(0.1 to 5.5)	(19.1 to 37.3)	(1.9 to 8.8)	(49.7 to 63.9)	(1.4 to 5.8)	(41.1 to 53.0)
Smoking in the past 3 months	0	23.3 *	1.1	46.2 *	0.7	38.5 *
		(15.7 to 33.1)	(0.3 to 4.5)	(39.1 to 53.4)	(0.2 to 2.8)	(33.0 to 44.4)
**Gambling (prevalence and 95% confidence interval)**						
Ever gambled (legal and illegal)	14.9	21.9	34.4	39.8	26.9	33.8
	(9.6 to 22.3)	(14.7 to 31.4)	(27.9 to 41.6)	(32.9 to 47.1)	(22.2 to 32.2)	(28.5 to 39.7)
Legal gambling in the past 12 months	3.8	8.3	25	24.9	16.9	19.4
	(1.6 to 9.1)	(4.2 to 14.7)	(19.3 to 31.7)	(19.2 to 31.8)	(13.1 to 21.4)	(15.1 to 24.5)
Illegal gambling in the past 12 months	6.5	12.1	13.4	17.2	10.8	15.5
	(3.3 to 12.3)	(6.9 to 20.4)	(9.3 to 19.0)	(12.3 to 23.5)	(7.8 to 14.7)	(11.7 to 20.3)
Gambling (legal and illegal) in the past 12 months	9.4	15.4	26.2	30.1	19.8	25.2
	(5.4 to 15.8)	(9.4 to 24.4)	(20.4 to 33.0)	(23.9 to 37.1)	(15.7 to 24.5)	(20.4 to 30.7)

* Significant difference between genders (*p*-value < 0.05).

**Table 3:** Associations between health-risk behaviours and injuries.

Health-Risk Behaviours	Health-Risk Behaviour Categories	Injuries Requiring Medical Attention aOR (95% CI), *p*-Value		Injuries Requiring Hospital Admission aOR (95% CI), *p*-Value		Injuries from Road Traffic Accidents Requiring Medical Attention aOR (95% CI), *p*-Value	
		Model 1	Model 2	Model 1	Model 2	Model 1	Model 2
Alcohol Use	Never	Reference	Reference	Reference	Reference	Reference	Reference
	Not in the past 3 months	1.51 (0.46 to 4.91), *p* = 0.491	1.05 (0.27 to 4.27), *p* = 0.941	2.51 (0.68 to 9.27), *p* = 0.165	2.12 (0.45 to 9.93), *p* = 0.337	2.06 (0.59 to 7.21), *p* = 0.257	1.58 (0.37 to 6.88), *p* = 0.538
	In the past 3 months but no heavy drinking in the past 30 day	3.51 (1.32 to 9.33), *p* = 0.012	2.20 (0.74 to 6.56) *p* = 0.157	2.20 (0.58 to 8.37), *p* = 0.248	1.68 (0.34 to 8.30), *p* = 0.524)	3.71 (1.27 to 10.9), *p* = 0.017	2.57 (0.73 to 9.14), *p* = 0.143
	Heavy drinking in the past 30 days	2.64 (0.87 to 7.97), *p* = 0.086	1.15 (0.34 to 3.89), *p* = 0.820	2.31 (0.52 to 10.2), *p* = 0.267	1.58 (0.31 to 8.08), *p* = 0.581	11.62 (0.40 to 6.51), *p* = 0.403	0.94 (0.19 to 4.69), *p* = 0.947
	*p*-value *	0.078	0.448	0.467	0.812	0.117	0.358
Smoking	Never	Reference	Reference	Reference	Reference	Reference	Reference
	Not in the past 3 months	7.67 (1.83 to 30.4), *p* = 0.004	6.50 (1.171 to 25.3), *p* = 0.014	5.51 (1.01 to 30.1), *p* = 0.049	4.69 (0.67 to 32.8), *p* = 0.119	5.29 (1.12 to 24.9), *p* = 0.035	4.22 (0.75 to 23.8), *p* = 0.103
	In the past 3 months	4.11 (1.19 to 14.3), *p* = 0.026	3.20 (0.81 to 12.6), *p* = 0.097	4.03 (1.03 to 15.8), *p* = 0.045	3.98 (0.78 to 20.0), *p* = 0.094	3.26 (0.89 to 11.9), *p* = 0.075	2.48 (0.58 to 10.5), *p* = 0.219
	*p*-value *	0.011	0.039	0.061	0.174	0.071	0.217
Gambling	Never	Reference	Reference	Reference	Reference	Reference	Reference
	Legal gambling in the past 12 months	2.59 (0.76 to 8.84), *p* = 0.128	2.23 (0.62 to 8.07), *p* = 0.220	–	–	1.68 (0.34 to 8.30), *p* = 0.522	1.52 (0.27 to 8.56), *p* = 0.637
	Illegal gambling in the past 12 months	1.27 (0.26 to 6.08), *p* = 0.762	1.05 (0.22 to 4.99), *p* = 0.954	1.84 (0.36 to 9.34), *p* = 0.463	1.69 (0.34 to 8.52), *p* = 0.523	1.42 (0.29 to 6.86), *p* = 0.661	1.21 (0.26 to 5.53), *p* = 0.803
	Both legal and illegal in the past 12 months	3.16 (1.13 to 8.83), *p* = 0.028	3.09 (1.05 to 9.05), *p* = 0.039	2.20 (0.63 to 7.62), *p* = 0.211	2.15 (0.64 to 7.22), *p* = 0.215	1.93 (0.57 to 6.50), *p* = 0.289	2.00 (0.52 to 7.71), *p* = 0.315
	*p*-value *	0.116	0.173	0.389	0.425	0.691	0.777

Model 1 was adjusted for age group, gender, education and living location; model 2 was adjusted for age group, gender, education, living location and all three health-risk behaviours; * *p*-value is for the overall association using an adjusted Wald test.

**Table 4:** Associations between the number of health-risk behaviours and injuries.

Number of Health-Risk Behaviours (Smoking, Drinking Alcohol and Gambling)	Injuries Requiring Medical Attention	Injuries Requiring Hospital Admission	Injuries from Road Traffic Accidents Requiring Medical Attention
	aOR (95% CI), *p*-Value	aOR (95% CI), *p*-Value	aOR (95% CI), *p*-Value
**0**	Reference	Reference	Reference
**1**	1.43 (0.49 to 4.16), *p* = 0.513	1.69 (0.47 to 6.14), *p*= 0.422	2.16 (0.65 to 7.20), *p* =0.209
**2**	3.81 (1.33 to 10.90), *p* = 0.013	2.02 (0.50 to 8.11), *p* = 0.322	4.19 (1.39 to 12.60), *p* = 0.011
**3**	13.8 (4.24 to 45.10), *p* < 0.001	8.02 (1.94 to 33.30), *p* = 0.004	8.20 (2.27 to 29.50), *p* = 0.001
*p*-value *	<0.001	0.037	0.008

Each injury outcome was modelled separately. Smoking was defined as having ever smoked and alcohol drinking was defined as ever having drunk alcohol. Gambling was defined as having gambled in the past 12 months. Each analysis was adjusted for age group, gender, education and living location; * *p*-value is for the overall association using an adjusted Wald test.
